# Characterization of human follicular thyroid cancer cell lines in preclinical mouse models

**DOI:** 10.1530/EC-15-0114

**Published:** 2016-03-01

**Authors:** Ashley N Reeb, Andrea Ziegler, Reigh-Yi Lin

**Affiliations:** Department of Otolaryngology, Head and Neck SurgerySaint Louis University School of Medicine, Saint Louis, Missouri, USA

**Keywords:** follicular thyroid cancer, orthotopic, thyroid, metastasis, mouse model

## Abstract

Follicular thyroid cancer (FTC) is the second most common type of thyroid cancers. In order to develop more effective personalized therapies, it is necessary to thoroughly evaluate patient-derived cell lines in *in vivo* preclinical models before using them to test new, targeted therapies. This study evaluates the tumorigenic and metastatic potential of a panel of three human FTC cell lines (WRO, FTC-238, and TT1609-CO2) with defined genetic mutations in two *in vivo* murine models: an orthotopic thyroid cancer model to study tumor progression and a tail vein injection model to study metastasis. All cell lines developed tumors in the orthotopic model, with take rates of 100%. Notably, WRO-derived tumors grew two to four times faster than tumors arising from the FTC-238 and TT2609-CO2 cell lines. These results mirrored those of a tail vein injection model for lung metastasis: one hundred percent of mice injected with WRO cells in the tail vein exhibited aggressive growth of bilateral lung metastases within 35 days. In contrast, tail vein injection of FTC-238 or TT2609-CO2 cells did not result in lung metastasis. Together, our work demonstrates that these human FTC cell lines display highly varied tumorigenic and metastatic potential *in vivo* with WRO being the most aggressive cell line in both orthotopic and lung metastasis models. This information will be valuable when selecting cell lines for preclinical drug testing.

## Introduction

Follicular thyroid cancer (FTC) is the second most frequently diagnosed thyroid cancer after papillary thyroid cancer (PTC). About 10,000 cases of FTC are diagnosed annually in the United States, comprising about 10–15% of all thyroid cancer cases. The majority of FTC patients have excellent prognoses when their cancers are caught early. However, the relative 5-year survival rate drops dramatically to about 50% for patients with advanced metastatic cancer who does not respond to standard treatments. More research is needed to determine the cause of the disease; to study tumor initiation, progression, and drug resistance; and to eventually develop new and improved drugs for this group of patients.

Human cell lines derived from tumors removed from thyroid cancer patients provide a useful model of thyroid disease mechanisms. Genetically defined cell lines with known driver mutations are particularly useful for clinicians and researchers striving to develop a more personalized approach to thyroid cancer therapy. Patient-derived thyroid cancer cell lines can also be used for *in vitro* toxicology studies, as well as *in vivo* studies in immunodeficient murine models to study cancer progression and drug responses in the tumor microenvironment. Previously, we and other laboratories developed an orthotopic thyroid cancer model that faithfully recapitulates the clinical characteristics of advanced thyroid cancer (1, 2, 3, 4). In addition, tail vein or intracardiac injection models have been developed to study the distant metastasis of human thyroid cancer cell lines (5).

In the current study, we characterize the tumorigenic and metastatic potential of a panel of three human FTC cell lines harboring various genetic mutations. The FTC-238 and TT2609-CO2 cell lines each has a *TP53* mutation commonly found in many cancers (6, 7). The WRO cell line has a *BRAFV600E* mutation. This mutation is the most common mutation found in PTC, but it is less common in FTC (8). We engineered these cell lines to express firefly *luciferase* to allow *in vitro* and *in vivo* tracking by bioluminescent imaging using an IVIS Spectrum. *In vivo* tumor growth was initiated by orthotopically injecting these cell lines into the thyroids of immunodeficient NSG (*NOD/ScidIl2rg-/-*) mice to initiate primary tumors. These cell lines were also injected into the lateral tail vein to evaluate their metastatic potential for lung colonization. Tumor progression and metastasis were evaluated by bioluminescent imaging weekly as well as postmortem histological analysis.

## Materials and methods

### Cell culture

The WRO cell line, established from the metastases of a female FTC patient, and TT2609-CO2 cell line, established from a 57-year-old male patient with FTC, were kindly provided by Dr John Copland (Mayo Clinic) with permission from Dr Guy J F Juillard (UCLA) for the WRO cell line and Dr Albert A Geldof (VU University Medical Center, The Netherlands) for the TT2609-CO2 cell line. The FTC-238 cell line, established from a lung metastasis of a 42-year-old male FTC patient, was purchased from ATCC. All cell lines have been confirmed by short tandem repeat (STR) analysis. The WRO cell line was cultured in DMEM/F12 (1:1) with 10% fetal bovine serum (FBS). The TT2609-CO2 cell line was cultured in RPMI1640 with 10% FBS. The FTC-238 cell line was cultured in DMEM/ F12 (1:1) with 5% FBS. The cultures were maintained in a humidified chamber in a 5% CO_2_:air mixture at 37°C.

### Quantitative real-time PCR

RNA was isolated from 1 × 10^6^ cells with the RNeasy Kit (Qiagen) and treated with RNase-free DNase (Qiagen). Two micrograms of RNA were reverse transcribed into cDNA using the Thermoscript First-strand Synthesis System (Invitrogen). The oligonucleotide sequences of the primers for *PAX8, TTF1 TSHR, TG,* and *TPO* have been published elsewhere (9, 10, 11). The mRNA levels were quantified in triplicate by quantitative real-time PCR on a ViiA7 PCR System (Applied Biosystems). Human *GAPDH* was used as the housekeeping gene during the amplification.

### Generation of *luciferase*-expressing cell lines

The cells were transfected with a pSIN-luc vector expressing the firefly *luciferase* gene (a gift from Dr Yasuhiro Ikeda of Mayo Clinic) to generate stable clones. A detailed description of the construction and transfection protocol has been published (12).

### Animals and ethics statement

Eight-week-old female NSG mice strains NOD.Cg*-Prkdc^scid^ Il2rg^tm1Wjl^/*SZJ were obtained from Charles River (Boston, MA, USA) and maintained under pathogen-free conditions at Saint Louis University Animal Facility. This study was carried out in strict accordance with the recommendations in the Guide for the Care and Use of Laboratory Animals of the National Institutes of Health. The protocol was approved by the Institutional Animal Care and Use Committee (IACUC) of Saint Louis University. All procedures were in accordance with institutional animal welfare guidelines, and all efforts were made to minimize suffering.

### Mouse tumor models

For orthotopic transplantation, 5 × 10^5^ cells were suspended in 10 μL Matrigel/RPMI or DMEM at a 1:1 dilution and injected into the right thyroid gland of NSG mice (1). The mice were killed when they reached humane endpoints including morbidity, immobility, unresponsiveness, recumbence, failure to eat or drink, and loss of more than 20% body weight. Tumors and adjacent tissues were collected and analyzed by hematoxylin and eosin (H&E) staining. In separate experiments, lung metastasis was experimentally induced by injecting 5 × 10^5^ cells into the lateral tail vein (13). The mice were imaged using IVIS weekly to monitor for metastatic tumor growth. At the time of killing the mice, lung tissues were harvested, fixed in 10% formalin, paraffin-embedded, and stained with H&E according to a standard protocol.

### Bioluminescent imaging

The anesthetized mice were injected with d-luciferin (150 mg/kg) and imaged with an IVIS Spectrum as described previously (14). The light emitted from the bioluminescent tumors was detected, digitized, and displayed, and the regions of interest from the displayed images were quantified as total photon counts per second (photons/s) using Living Image software (Caliper Life Sciences).

## Results

### Real-time PCR analysis of thyroid-specific genes

**Figure 1.**
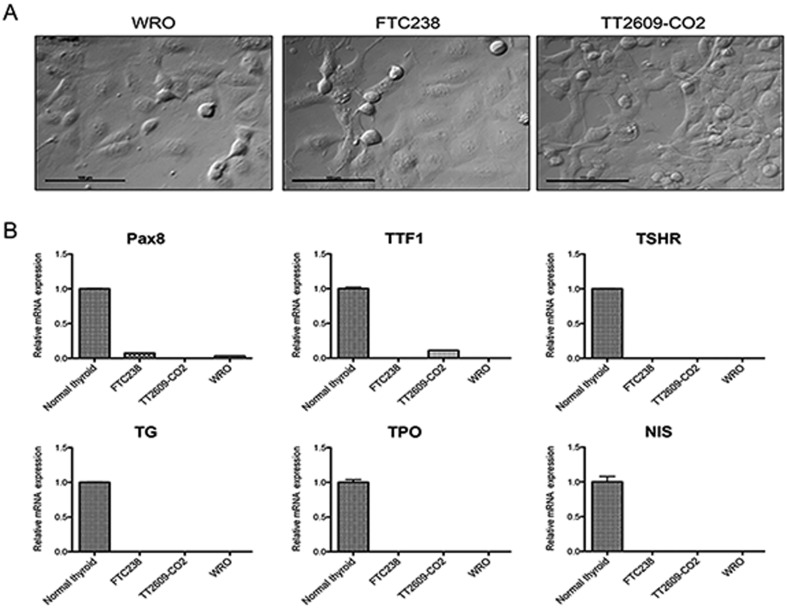
Morphologies and gene expression analysis of human FTC cell lines. (A) Phase-contrast microscopy images of WRO, FTC-238, and TT2609-CO2 cell lines. (B) Quantitative real-time PCR analysis of *PAX8, TTF1, TSHR, TG, TPO,* and *NIS. Y*-axis, relative mRNA expression level compared with normal human thyroid. Human *GAPDH* was used as the housekeeping gene during the amplification for the normalization of the above gene expression data. The results are expressed as mean ± s.e.m. of two independent experiments with three parallels.

Three human FTC cell lines (WRO, FTC-238, and TT2609-CO2) were validated and obtained from the sources indicated in the Materials and methods section. The mutational characteristics of these human FTC cell lines, based on published reports (7, 8, 15), are summarized below. FTC-238 and TT2609-CO2 each has a *TP53* mutation, and WRO has a *BRAF*V600E mutation. None of the cell lines have mutations in *HRAS, NRAS, KRAS, PI3KCA*, or *PTEN*. Phase-contrast images of the three FTC cell lines are depicted in Fig. 1A. Next, we used quantitative real-time PCR to examine the expression of thyroid-specific genes in these three cell lines. We found that FTC-238 and WRO express *PAX8*, a thyroid transcription factor, whereas TT2609-CO2 expresses thyroid transcription factor 1 (*TTF1*). However, unlike normal human thyroid, none of these cell lines had detectable levels of mRNA for the thyroid differentiation markers thyroglobulin (*TG*), thyroperoxidase (*TPO*), sodium-iodide symporter (*NIS*), or the receptor for thyroid stimulating hormone (*TSHR*) (Fig. 1B), confirming the dedifferentiated state of these human FTC cell lines.

### Generation of stable *luciferase*-expressing human FTC cell lines

**Figure 2.**
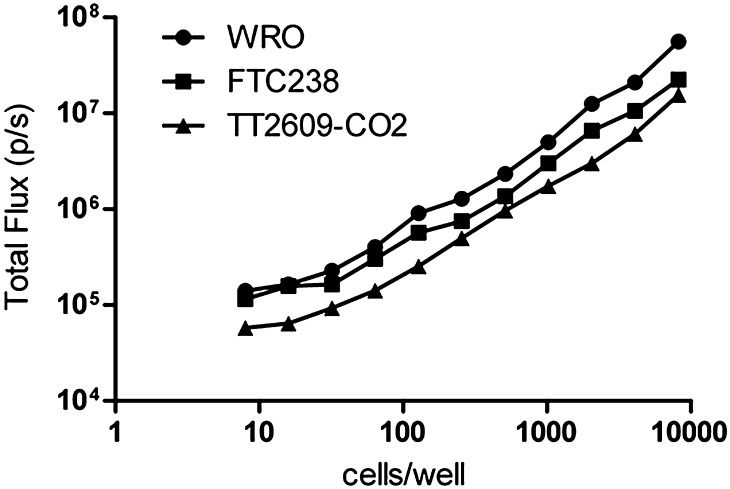
*In vitro* bioluminescence of WRO, FTC-238, and TT2609-CO2 cell lines. Stable clones of WRO, FTC-238, and TT2609-CO2 cells expressing *luciferase* were serially diluted. Luciferin substrate was added to each well 10 min before imaging and the plate was imaged to obtain total flux (p/s) per cell line using IVIS Spectrum. The wells with media but not cells, or cells alone without luciferin, were included as controls. Note that the level of *luciferase* expression was proportional to the number of cells seeded. The results are expressed as mean ± s.e.m. of two independent experiments with three parallels.

To generate *luciferase*-expressing cell lines suitable for *in vitro* and *in vivo* live imaging, WRO, FTC-238, and TT2609-CO2 cells were transfected with a pSIN-luc vector encoding a firefly *luciferase* gene, and three stable clones from each cell line were selected for further analysis. The levels of luciferase activity in serially diluted cell cultures were quantified *in vitro* using IVIS in complete media supplemented with luciferin. The results show that luciferase activity was proportional to the number of seeded cells (from 10 to 10,000) for all cell lines (Fig. 2).

### Tumorigenesis analysis using an orthotopic mouse model of thyroid cancer in immunodeficient mice

**Figure 3.**
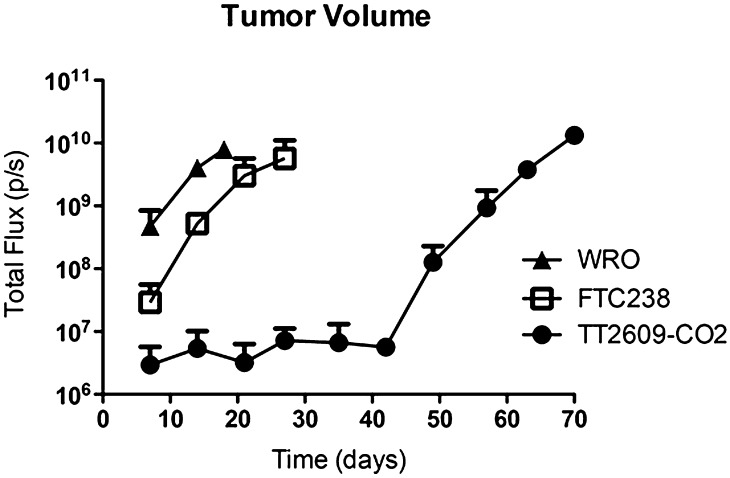
WRO-derived tumors grew faster than FTC-238 and TT2609-CO2-induced tumors in a murine orthotopic model. Luciferase-tagged WRO, FTC-238, or TT2609-CO2 cells were injected into the thyroids of NSG mice, and tumor initiation and growth were monitored *via* bioluminescent live imaging weekly using IVIS Spectrum. Bioluminescent tumor signals were quantified as total flux (p/s) using Living image software and plotted against the days since tumor cell injection. The results are expressed as mean ± s.e.m. (*n* = 2 per group).

To explore whether the FTC cell lines could initiate tumors *in vivo*, we used an orthotopic thyroid cancer model with weekly live imaging in immunodeficient mice to assess tumor initiation and progression (1). In this model, 5 × 10^5^ *luciferase*-expressing WRO, FTC-238, or TT2609-CO2 cells were orthotopically injected into the right thyroid of 8-week-old female NSG mice (*n* = 2 per cell line). Tumor initiation and growth were monitored weekly *via* bioluminescent live imaging using IVIS. Notably, WRO-derived tumors were more aggressive and invasive and grew two to four times faster than tumors arising from the FTC-238 and TT2609-CO2 cell lines. Tumor volume, assessed as total flux (photon counts per second), revealed that mice injected with WRO cells rapidly developed primary tumors within 7 days of injection. These mice survived a total of 14–18 days, whereas mice injected with FTC-238 cells survived a total of 27–29 days. In contrast, mice injected with TT2609-CO2 cells experienced significantly delayed onset of tumors and the tumors grew more slowly than those arising from WRO or FTC-238 cells. These mice survived a total of 57–70 days (Fig. 3).

**Figure 4.**
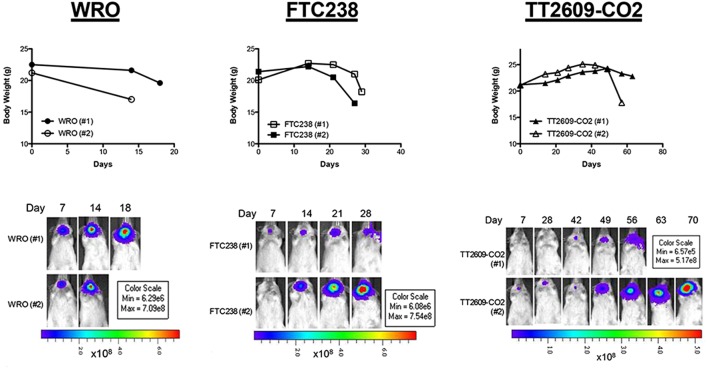
Time course of body weight and *in vivo* bioluminescence. Upper panel shows body weights of mice injected with WRO, FTC-238, or TT2609-CO2 cell lines. Lower panel shows primary thyroid tumors resulting from injection of WRO, FTC-238, or TT2609-CO2 cells in the orthotopic models as visualized by bioluminescent IVIS imaging. All mice (*n* = 2 per group) were reimaged using the same setting of the IVIS throughout the study.

An important feature of the orthotopic thyroid cancer model, which recapitulates advanced stage thyroid cancers, is that cancer cells are directly injected into the thyroid. As a result, aggressive tumor invasion occurs in nearby vital organs such as the trachea and esophagus. The subsequent compression of these organs leads to significant weight loss: a phenomenon we confirmed here with all three FTC cell lines (Fig. 4). For humane reasons, animals were killed when they had lost up to 20% of their body weight, an endpoint that was often accompanied by signs of unresponsiveness and failure to eat or drink. According to these criteria, one of the WRO-injected mice was killed 14 days after tumor cell injection, while the other mouse survived a total of 18 days (Fig. 4). FTC-238-injected mice were killed at day 28, but mice injected with TT2609-CO2 cells experienced a delayed onset of tumors. These mice continued to gain weight until day 49, when they began losing weight. One of these animals experienced a rapid decline in body weight beginning at day 56, while the other mouse survived a total of 70 days (Fig. 4).

**Figure 5.**
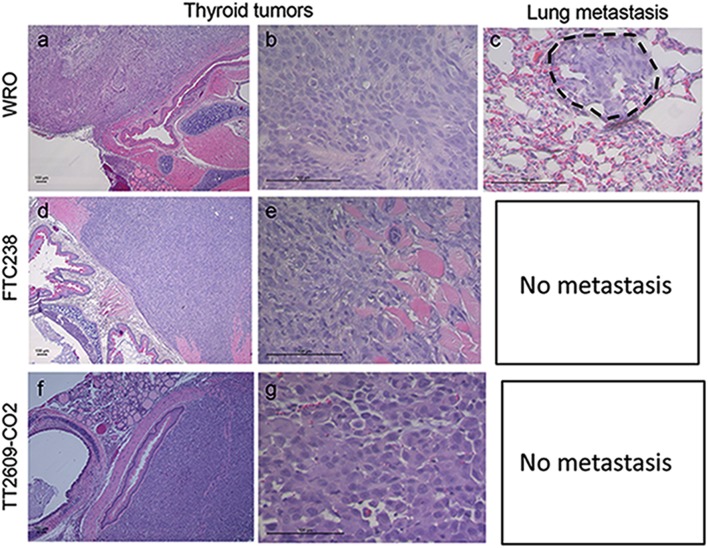
Representative histology images of thyroid tumors and lung metastases in a murine orthotopic model. H&E stained sections of all mice orthotopically injected with WRO, FTC-238, or TT2609-CO2 cell lines show the primary tumor with local invasion to trachea and esophagus (A,D,F). The middle panel shows higher magnification (200x) of tumors from each cell line (B,E,G). Note that mice injected with WRO also developed a lung micrometastasis whereas mice injected with FTC-238 or TT2609-CO2 cells did not ©. Scale bar, 100 mm.

Postmortem histological analysis showed that all mice receiving WRO, FTC-238, and TT2609-CO2 cells developed thyroid tumors, with subsequent invasion into the trachea, esophagus, and surrounding muscles. Notably, mice injected with WRO cells also exhibited tumor cell invasion in their lung tissue, but mice injected with FTC-238 or TT2609-CO2 cells did not (Fig. 5). These observations demonstrate that WRO-derived tumors grow two to four times faster than tumors arising from FTC-238 and TT2609-CO2 cell lines in our orthotopic model, and show that WRO is the only cell line examined that can invade the lung and develop micrometastases under these conditions.

### Lung metastasis of FTC cell lines in a tail vein injection model

**Figure 6.**
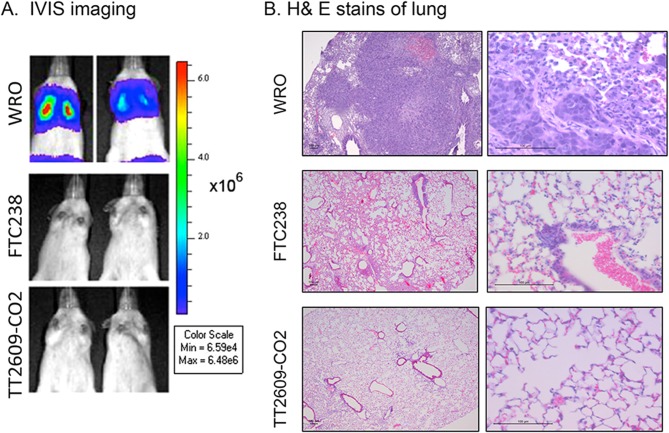
WRO cells develop lung metastases in the tail vein injection model. Lung metastases were experimentally induced by tail vein injection of 5 × 10^5^ cells. (A) Representative bioluminescent images of mice (*n* = 2 per group) injected with WRO, FTC-238, or TT2609-CO2 cells taken at day 35. (B) H&E stained sections revealed tumor cells metastasized to the alveoli of the lung in mice injected with WRO cell line. Left panel, 50x magnification; right panel, 200x magnification. Scale bar, 100 mm.

To ensure that our orthotopic model did not underestimate the metastatic potential of FTC cell lines, perhaps because the mice may have reached the point of humane killing before lung metastasis, we established an artificial metastasis model by directly injecting tumor cells intravenously into the mouse tail vein. This technique allows us to more clearly evaluate the metastatic potential of each cell line. In this model, 5 × 10^5^ cells were injected into the lateral tail vein. IVIS imaging showed that all mice injected with WRO cells exhibited rapid and aggressive growth of bilateral lung metastases (*n* = 2). In contrast, all mice injected with FTC-238 or TT2609-CO2 cells remained free of tumors 35 days after injection (Fig. 6A). Additional H&E staining of the isolated lung tissues of the mice injected with WRO cells confirmed tumor cells metastasized to the alveoli of the lung, and that those mice injected with FTC-238 or TT2609-CO2 cells did not develop lung metastasis (Fig. 6B).

## Discussion

The aim of this study was to assess a panel of three human FTC cell lines for their tumorigenic and metastatic potential in xenotransplantation assays in immunodeficient mice. Our data show that all three human FTC cell lines form tumors in immunodeficient NSG mice with a 100% take rate. In addition, the three cell lines display highly varied *in vivo* tumor growth trends; WRO was the most aggressive cell line in both our orthotopic and tail vein injection models.

For many years, investigators have relied on immunodeficient mice such as NOD/SCID mice or athymic nude mice for basic and preclinical research because they do not reject tumor cells transplanted from humans or other species. Athymic mice have no functional T cells. NOD/SCID mice have both T-cell and B-cell deficiencies, but they retain cytotoxic activity due to NK cells and macrophages. As a result, they may not produce high take rates in xenograft studies. A recent report evaluating primary thyroid tumor growth efficiency following orthotopic implantation and intracardiac injection in athymic nude mice has shown a highly variable take rate, ranging from 0 to 100% in 11 thyroid cancer cell lines (4). Such variability raises a concern as to whether the athymic nude mice can be used to adequately evaluate the tumorigenic potential of a given thyroid cancer cell line and whether they are a good choice for preclinical drug testing. In this study, we used NSG mice to evaluate the tumorigenic potential of three human FTC cell lines. This NSG mouse strain lacks mature T cells and B cells and is also deficient for *Il2, Il4, Il7*, and *Il12*, which are required for NK cells and innate immunity responses (16). Therefore these mice are more severely impaired than the commonly used NOD/SCID or athymic nude mice and they represent a gold standard for cancer research due to their superior xenografting capability (17, 18). We have previously used the orthotopic and tail vein injection models in NSG mice to study the anaplastic thyroid cancer cell (ATC) lines THJ-11T and THJ-16T. In these studies, we achieved a 100% tumor take rate (11, 13). We have also used NSG mice to confirm the presence of a rare subpopulation of cancer stem cells in the ATC cell lines (14). In this study, all three human FTC cell lines developed tumors in the orthotopic model with a 100% take rate. Recently, Zhang and coworkers used the same mouse strain and achieved a 100% take rate for all eight thyroid cancer cell lines examined (19). Together, these studies emphasize the importance of mouse strain choice in evaluating tumorigenic potential of a given thyroid cancer cell line in order to increase the efficiency and reduce the costs of preclinical drug testing.

In this study, we found that WRO cell line, which has a *BRAFV600E* mutation, is the most tumorigenic of the three human FTC cell lines tested. Activating mutations in *BRAF*, a 766-amino acid serine/threonine-specific protein kinase, have been detected in a wide range of human malignancies. The presence of *BRAF* mutations is significantly associated with advanced thyroid cancers with metastasis and poor prognoses. Therefore our data support the proposition that the *BRAFV600E* mutation renders the WRO cell line particularly aggressive. Because the *BRAFV600E* mutation, which is commonly associated with high-risk clinicopathologic characteristics of patients with PTC, is less common in FTC (8), the origin of the WRO cell line has been questioned. However, several investigators have demonstrated with STR profiling analyses that the WRO cell line is not contaminated (7, 15).

In addition to driver mutations, a number of other mechanisms may also contribute to the aggressiveness of FTC cell lines. Schmutzler and coworkers reported an inhibitory effect of all-*trans* retinoic acid (RA) on the growth of FTC-133 and FTC-238 cell lines (20). Both FTC-133 and FTC-238 were derived from the same patient, a 42-year-old male with FTC. Specifically, FTC-133 was established from a primary tumor, whereas FTC-238 from a lung metastasis. In *in vivo* studies, pretreatment of FTC-133 and FTC-238 with all-*trans* RA resulted in a reduced tumor growth after xenotransplantation onto adult athymic nude rats (20). The authors suggested that tumor-specific RA receptors might contribute to the pathogenesis and tumor progression of human thyroid cancer cell lines. In another study, Hoffmann and coworkers demonstrated a major differential expression pattern of integrin receptor molecules (IRM) in differentiated (FTC and PTC) and undifferentiated (ATC) thyroid cancer cell lines. Through cell substratum adhesion assays, they noted that the metastatic thyroid cancer cell lines express high levels of integrins α1-6 and β1 and show strong attachment to extracellular matrix (ECM) proteins including collagen I, collagen IV, laminin, and fibronectin (21). It is conceivable that these integrins may contribute to the development of metastatic disease.

In summary, our work demonstrates that these human FTC cell lines display highly varied tumorigenic potential, with WRO being the most aggressive cell line in both *in vivo* models. This new information will be useful for selecting cell line for future pre-clinical drug testing and for basic research aiming at delineating the molecular basis for thyroid cancer pathogenesis.

## Declaration of interest

The authors declare that there is no conflict of interest that could be perceived as prejudicing the impartiality of the research reported.

## Funding

This work was supported in part by the National Institutes of Health Grant DK068057 (to R.Y.L.).
